# Expression of acetylated histones H3 and H4 and histone deacetylase enzymes HDAC1, HDAC2 and HDAC6 in simple mammary carcinomas of female dogs

**DOI:** 10.3389/fgene.2023.1257932

**Published:** 2023-11-09

**Authors:** Igor Luiz Salardani Senhorello, Oscar Rodrigo Sierra Matiz, Isabela Cristina Canavari, Giovanny Vargas Hernandez, Letícia Abrahão Anai, Roberto Andrés Navarrete Ampuero, Josiane Moraes Pazzini, Cibele Maria Prado, Flavio Vieira Meirelles, Rosemeri de Oliveira Vasconcelos, e Mirela Tinucci-Costa

**Affiliations:** ^1^ Department of Veterinary Clinic and Surgery, Faculty of Agricultural and Veterinary Sciences, Universidade Estadual Paulista, Jaboticabal, SP, Brazil; ^2^ Department of Veterinarry Medicine, Universidade Vila Velha-UVV, Vila Velha, Espírito Santo, Brazil; ^3^ Department of Animal Health, Universidad Nacional de Colombia, Bogotá, Colombia; ^4^ Autonomous Veterinarian, São Paulo, Brazil; ^5^ Department of Veterinary Medicine, Faculty of Animal Science and Food Engineering, Universidade de São Paulo-USP, Pirassununga, São Paulo, Brazil

**Keywords:** epigenetic, prognosis, dogs, mammary carcinomas, histones

## Abstract

Histone deacetylation is an important mechanism involved in human breast cancer tumorigenesis and recent veterinary oncology studies also demonstrate a similar relationship in some canine neoplasms. The use of HDAC inhibitors *in vitro* and *in vivo* has demonstrated antitumor action on several strains of human and animal cancers. The present study aims to correlate the expression of H3K9Ac, H4K12Ac, HDAC1, HDAC2 and HDAC6 in simple mammary carcinomas in dogs with clinicopathological parameters and overall survival time. To this end, 61 samples of simple breast carcinomas were analyzed by the immunohistochemistry technique with subsequent validation of the antibodies by the Western Blot technique. The expressions obtained via a semi-quantitative way were categorized by assigning scores and classified into high or low expressions according to the given score, except for HDAC6, when the marking percentage was considered and subdivided into high and low expressions using the median value. For statistical analysis, the chi-square test or Fisher exact test were used as univariate analysis and correspondence analysis as a multivariate test, in addition to the Kaplan-Meier survival analysis. In the studied samples, the highest frequencies were determined for the high expression proteins H4K12Ac (88.5%), HDAC2 (65.6%) and HDAC6 (56.7%) and the low expression proteins H3K9Ac (73.8%) and HDAC1 (54.1%). An association between the low expression of HDAC1 and the presence of lymph node metastasis (*p* = 0.035) was indicated by univariate analysis while the high expression of HDAC1 was associated with favorable prognostic factors, such as the absence of lymph node metastasis and low mitotic index by multivariate analysis. Also, by multivariate analysis, the low expression of HDAC6 was correlated with the low expression of Ki67, smaller tumors, and better prognosis factors as well. Protein expression was not correlated with patients’ overall survival time (*p* > 0.05). The high expressions of HDAC2 and HDAC6 in mammary carcinomas in female dogs may be useful information for research involving therapeutic targets with iHDACs since their inhibition favors hyperacetylation and transcription of tumor suppressor genes.

## Introduction

Epigenetic alterations have become an important focus of oncological research since histone acetylation and DNA methylation profiles were correlated with tumorigenesis (Oronsky et al., 2014; Lu et al., 2020; Moufarrij et al., 2020).

Histone deacetylases (HDACs) are enzymes responsible for removing the acetyl groups of histones, leading to a hypoacetylation of these proteins and a greater interaction with the DNA, keeping it compact. Thus, accessibility to coding regions is impaired, leading to the suppression of transcriptionally active genes (Jenuwein and Allis, 2001; Muller and Prado, 2008). Therefore, histone deacetylation caused by the overexpression of HDACs leads to cell proliferation, cell migration, and inactivation of tumor suppressor genes, thus, favoring tumorigenesis (Nakagawa et al., 2007; Xu et al., 2007; Uehara et al., 2012; Uehara et al., 2012; Eckschlager et al., 2017).

Among the histones whose amino-terminal regions can be modified, H3 and H4 are the most important (Elsheikh et al., 2009). As for HDACs, there are at least 18 known enzymes (Cheung et al., 2000; Seto and Yoshida, 2014), and HDACs 1, 2 and 6 are among the most studied in human breast cancer (Zhang et al., 2004; Krusche and Wulfing, 2005; Müller et al., 2013; Derr et al., 2014; Zhao et al., 2016).

Recent studies demonstrate that using HDAC inhibitors (iHDACs) *in vitro* and *in vivo* induces apoptosis, decreases cell migration, and impairs cell differentiation in several human and animal cancer lineages, exerting important antitumor activity (Murahari et al., 2017; Eto et al., 2019; Lu et al., 2020; Moufarrij et al., 2020). Histone modifications and aberrant expressions of HDACs have been linked to prognostic factors in human and animal tumors (Bryan et al., 2009; Elsheikh et al., 2009; Müller et al., 2013; Eto et al., 2019; Matiz, 2019; Sena et al., 2022).

Mammary neoplasm in female dogs is the main neoplasm among unneutered female dogs, with mammary carcinomas being the most common histological type, similar to the histological data on woman breast cancer (Shafiee et al., 2013; Cassali et al., 2014; Sorenmo et al., 2020; Sorenmo et al., 2020). Furthermore, the publication of the canine genome has shown that dogs are an experimental model for several diseases affecting humans while the biological behavior of cancer in humans and dogs is similar. In this regard, breast tumors are particularly similar between the two species (Lindblad-Toh et al., 2005; Ranieri et al., 2013; Shafiee et al., 2013).

Several established prognostic factors help determine the clinical approach to canine patients with breast tumors. However, despite having a neoplasm with the same clinical-pathological characteristics, the tumors in some animals do not evolve in the same way, suggesting the existence of different mechanisms involved in tumorigenesis (Cavalcanti et al., 2006; Santos et al., 2013; Cassali et al., 2014; Rasotto et al., 2017).

In human medicine, the knowledge of neoplasm epigenetic alterations allows using the histone deacetylase inhibitors (iHDACs). These iHDACs have already been approved for use in cutaneous lymphomas while clinical trials for use in breast tumors are advanced (Weichert, 2009; Guerriero et al., 2017). Furthermore, knowing the relationship between epigenetic changes and prognosis favors a better understanding of the biological behavior of the tumor, thus allowing to select the patients who would benefit from individualized treatments (Muller et al., 2013; Qiao et al., 2018; Eto et al., 2019; Matiz, 2019).

Considering the similarity in many aspects between mammary tumors in women and female dogs, this study aimed to analyze the expression of H3K9Ac, H4K12Ac, HDAC1, HDAC2 and HDAC6 in simple mammary carcinomas in dogs and to correlate the clinicopathological variables and the prognosis with the expression of these proteins.

## Material and methods

### Patient selection and sampling

This study was approved by the Ethics Committee on Animal Experimentation of the College of Agrarian and Veterinary Sciences of UNESP, in Jaboticabal (Protocol no 016384/17). Additionally, the dog owners were requested to sign a guidance and informed consent form.

This study investigated sixty-one female dogs selected without preference for race or age that were affected by mammary neoplasms and treated at the Veterinary Obstetrics Service and the Oncology Service of the Veterinary Hospital of FCAV/UNESP, in Jaboticabal, SP. Tumor staging was performed in all dogs by detailed physical examination, as well as laboratory and imaging tests.

To standardize, only female dogs diagnosed with simple carcinoma, including the papillary, tubular and solid subtypes, were used. Only one nodule was sampled per dog; when several nodules were present, the nodule associated with the worst prognosis was selected after grade and clinical staging (Rasotto et al., 2017). Patients with mixed tumor carcinomas, carcinomas of special subtypes and breast sarcomas (Cassalli et al., 2014), as well as distant metastasis at the time of diagnosis were excluded from the study. However, patients that developed metastasis during follow-up and after mastectomy remained in the study.

Before sampling, all patients underwent general anesthesia following the protocol used at the veterinary hospital. Breast tumors were collected during total, uni or bilateral mastectomy, according to the individual indication (Cassali et al., 2014), during which the regional lymph node (inguinal and/or axillary) was removed for histopathological evaluation.

All samples collected were fixed in 10% buffered formalin for 24–48 h and then stored in 70% alcohol until processing. Subsequently, the sample processing consisted of embedding the tissue in paraffin and cutting to a thickness of 4 µ for histopathological analysis (Cassali et al., 2104) and the immunohistochemistry technique.

### Evaluation of clinicopathological parameters

Clinical-pathological data of each animal such as race, age, histological type, tumor size, histological grade, regional lymph node metastasis, mitotic index, and overall survival (OS) were evaluated individually. Regarding tumor grade, the groups were divided into grades I, II and III, following the histopathological classification adopted by Elston and Elis (1998).

Tumor size was obtained by measuring the largest diameter using a digital caliper and expressed in centimeters (cm) (Yamagami et al., 1996). The determination of the mitotic index followed the methodology previously employed by Santos et al. (2013), wherein tumors were categorized as having a low mitotic index (up to 9 mitotic figures in 10 high-power fields (HPF)) and moderate to high mitotic index (>9 mitotic figures in 10 HPF). The examination was conducted using a Novel microscope (BM2100) with a ×40 objective.

### Validation of antibodies by the Western blot technique

Of the studied dogs, nine samples of simple mammary carcinomas were selected and homogenized at 4°C in RIPA buffer (50 mM Tris HCl, pH 8.0, 150 mM NaCl, 1% NP-40, 0.5% sodium deoxycholate, 0.1% SDS), pH 7.4 and protease inhibitor cocktail (# P8310 - Sigma). The homogenate was centrifuged at 14,000 g and 4°C for 20 min. The protein concentration of the supernatant was determined by the Lowry method using bovine serum albumin (BSA) as a standard. The supernatant was mixed with sample buffer (20% glycerol, 125 mM Tris-HCl, 4% SDS, 100 mM dithiothreitol, 0.2% bromophenol blue, pH 6.8), the mixture was boiled and submitted to electrophoretic analysis in 10% polyacrylamide gel (SDS-PAGE), and the proteins transferred from the gel to the nitrocellulose membranes.

Membranes were blocked with 5% albumin for 4 h and incubated overnight at 4°C with the primary antibodies acetyl Histone H4 [Ac-Lys12] (1:1000, anti-rabbit, Sigma-Aldrich), HDAC1 (1:1000 μg, anti-rabbit, Sigma-Aldrich), HDAC2 (1:2000, anti-mouse, SigmaAldrich), acetyl Histone H3 [Ac-Lys9] (1:1000, anti-mouse, Sigma Aldrich) and HDAC6 (1:3000, anti-rabbit, Sigma-Aldrich). GAPDH (1:1000, Cell Signaling, Danvers, MA, USA) and β-actin (1:5000, Santa Cruz Biotechnology, Santa Cruz, CA, USA) were used as loading controls. For detection, the secondary antibody conjugated with peroxidase was used and visualized using the ECL reagent. Gel documentation and signal quantification were obtained using the Bio-Image Analysis of the Molecular Imager ChemiDoc XRS system (Bio-Rad, Richmond, CA, USA). Results were normalized using GAPDH to H3Ac and HDAC6, as well as B-actin to H4Ac, HDAC1, and HDAC2. The marking bands were evidenced, and the samples were quantified for validating the antibodies in canine tissue.

### Immunohistochemical reaction

Immunohistochemical reactions were used to detect the acetylated histones, H3 lysine 9 (H3K9Ac) and H4 lysine 12 (H4K12Ac), histone deacetylase enzymes HDAC1, HDAC2, HDAC6, and cell proliferation marker Ki67 in neoplastic tissue samples. The reactions with Ki67 (MIB-1 clone, Dako; 1:100) followed the protocol described previously by Sierra et al. (2018).

For the other antibodies, the detection method employed a commercial polymer system (Novolink DS polymer, Leika Biosystems), as described in Table 1. A human breast sample, kindly provided by the VETPAT laboratory (Campinas, SP), was used as a control of the reactions, and as a negative control, the primary antibodies were replaced by antibody diluents. Samples were counterstained with Harris hematoxylin.

**TABLE 1:** Antibodies used in the immunohistochemistry technique for the expression of acetylated histones and deacetylase enzymes.

Antibody^a^	Clone	Origin	Type	Dilution	Antigenic recovery
Anti-Acetyl Histone H3 (Ac-Lys9)	AH3-120	Rat	Monoclonal	1:100	Citrate Buffer (pH 6)
Anti-Acetyl Histone H4 (Ac-Lys12)	SAB4200353	Rabbit	IgG fraction	1:200	Citrate Buffer (pH 6)
Anti-HDAC1	AV38530	Rabbit	IgG fraction	1:75	EDTA ± Buffer (pH 9)
Anti-HDAC2	HDAC2-62	Rat	Monoclonal	1:500	Citrate Buffer (pH 6)
Anti-HDAC6	AV31451	Rabbit	Isolated	1:500	Citrate Buffer (pH 6)

^a^ Sigma-Aldrich; ± Ethylenediamine tetra acetic acid; All antibodies were incubated for 2 hours at 22°C.

### Interpretation of immunohistochemistry results

The nuclear expression in sections immunostained for H3K9Ac, H4K12Ac, HDAC1 and HDAC2 was confirmed by the presence of diffuse brown tones in the nuclei of cells of the simple mammary carcinoma samples, as observed in the positive controls.

The results of the immunohistochemical reactions were interpreted based on the adopted marking scores. For this purpose, the following protocol was adopted: five random fields (from each sample) were photographed at 400× magnification, using a Novel microscope (BM2100) coupled to a Bioptika camera (CMOS-HD). Next, 100 cells per field were manually counted using the “Cell Counter” tool of the ImageJ software (v. 1.44p.), and subsequent calculation of the arithmetic means. The cell field was ranked by percentage of positive cells (negative = 0; 1%–25% = 1; 26%–50% = 2; 51%–75% = 3; 76%–100% = 4) and staining intensity. The latter was evaluated blindly by two observers (negative = 0; weak = 1; moderate = 2, and intense = 3). Finally, the two parameters (percentage and marking intensity) were multiplied while the final marking score obtained was classified as low (0–6 score) and high (8–12 score) expression and then submitted to statistical analysis (Zhao et al., 2016).

However, a different method was used for the reactions with the HDAC6 antibody since the cytoplasmic expression made it impossible to count individual cells. The positive expression was characterized by observing and classifying the shades of brown in the cell cytoplasm, considering the marked area. The total percentage of area positive for HDAC6 was calculated by obtaining images of five random fields amplified 400 times using a Novel microscope (BM2100) coupled to a Bioptika camera (CMOS-HD) and the ImageJ software (v. 1.44p), as previously described (Withers et al., 2019). First, the total cellular area was determined and measured for each image while excluding non-cellular areas manually. Subsequently, a positive area was manually selected to highlight only positive cells. The selected area was then measured, and the result was divided by the total cellular area in the image and multiplied by 100 to calculate the percentage for each image. A final percentage was estimated from the average of the five images for each sample. The median of the positive marking values was the cut-off point established to classify enzyme expression in the tissue as low or high.

To evaluate the reactions with the Ki67 antibody, five fields at ×400× magnification and containing areas with higher proportions of positive cells were photographed, using the same imaging systems described previously. A total of 200 cells were manually counted in each sample, totaling 1000 cells (De Matos et al., 2006; Kadthur et al., 2011). The percentage of positive cells was obtained from this value and, the median value of all samples was used to classify into high and low expression.

### Statistical analysis

Contingency tables were prepared to display the frequency distribution of the categorical variables (histological grade, histological type, tumor size, presence of metastasis in lymph nodes, mitotic index, and Ki67 expression) and continuous variables (H4K12Ac, H3K9Ac and HDAC 1, 2 and 6) classified into high and low expression. Frequencies were compared using either the Pearson chi-square test or Fisher exact test. Additionally, multivariate correspondence analysis of the associations between the expression of acetylated histones (H3K9Ac and H4K12Ac) and deacetylase enzymes (HDAC 1, 2, and 6) and clinicopathological variables in simple mammary carcinomas in female dogs was performed, and a perceptual map was generated.

The survival curves were determined using the Kaplan-Meier survival analysis for the overall survival time and compared by the Long-rank test. Overall survival (OS) was defined as the time elapsed from the day of diagnosis to the date of death. The dogs that remained alive after 3 years, were classified as censored; the medical record data was updated at the end of the experimental period via a telephone call to the dog owner. The R, SPSS and Statistica software were used at a 5% significance level.

## Results

Papillary carcinoma (Cassali et al., 2014) was diagnosed with the highest frequency (27/61; 44.26%), followed by tubular carcinoma (24/61; 39.34%) and solid carcinoma (10/61; 16.40%). Most of the patients were mixed breed animals (20/61; 33%), aged between 5 and 15 years (mean 10 ± 2.42) (Table 2). The clinicopathological variables of female dogs with simple mammary carcinomas are shown in Table 2.

**TABLE 2:** Clinicopathological variables of simple mammary carcinoma observed in dogs.

Clinical variable		Total dogs	Percentage (%)	Mean (interval)
	Mixed Breed	20	33	
	Poodle	6	10	
	York Shire Terrier	6	10	
Breed	Pinscher	5	8	
	Pitbull	4	6	
	Other	20	33	
Age (years)				10 (5-15)
Tumor size (major axis)	<3 cm	42	69	
	≥ 3 cm	19	31	
Histological grade	I	28	46	
	II	21	34	
	III	12	20	
Mitotic index/10 HPF	0–9	40	66	
	>9	21	34	
Ki67*	<18%	32	52	
	>18%	29	48	

MB: mixed breed; <3 cm: smaller than 3 cm; ≥ 3 cm: larger or equal to 3 cm; HPF: high power field; *Ki67: cutoff value given by the median.

The clinicopathological variables on necrosis and ulceration were not included in the table due to the low frequency observed in this study.

### Validation of antibodies by the Western blot technique

**FIGURE 1:**
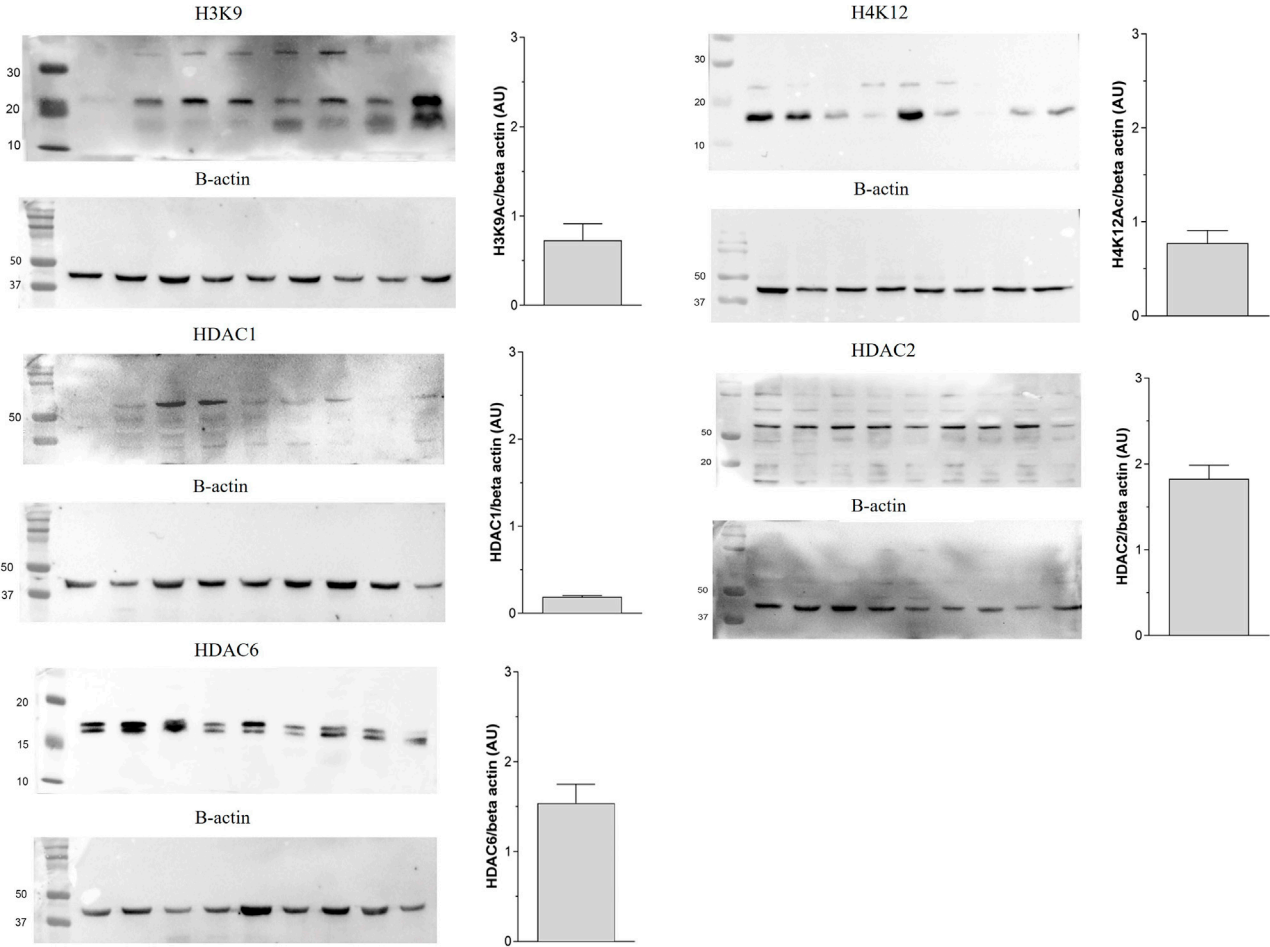
Validation of protein expression of acetylated histone (H3K9Ac and H4K12Ac) and histone deacetylases (HDAC1, HDAC2 and HDAC6) by Western Blot technique in simple mammary carcinomas of female dogs.

The protein expression detected through the Western Blot technique in nine samples of canine simple mammary carcinomas confirmed the immunoreactivity of human antibodies with canine tissue, thereby validating the immunohistochemical reactions. Furthermore, these expressions were quantified as part of the technique’s validation process (Figure 1).

### Immunohistochemical expression of acetylated histones and histone deacetylase enzymes and correlation with clinicopathological variables

**FIGURE 2:**
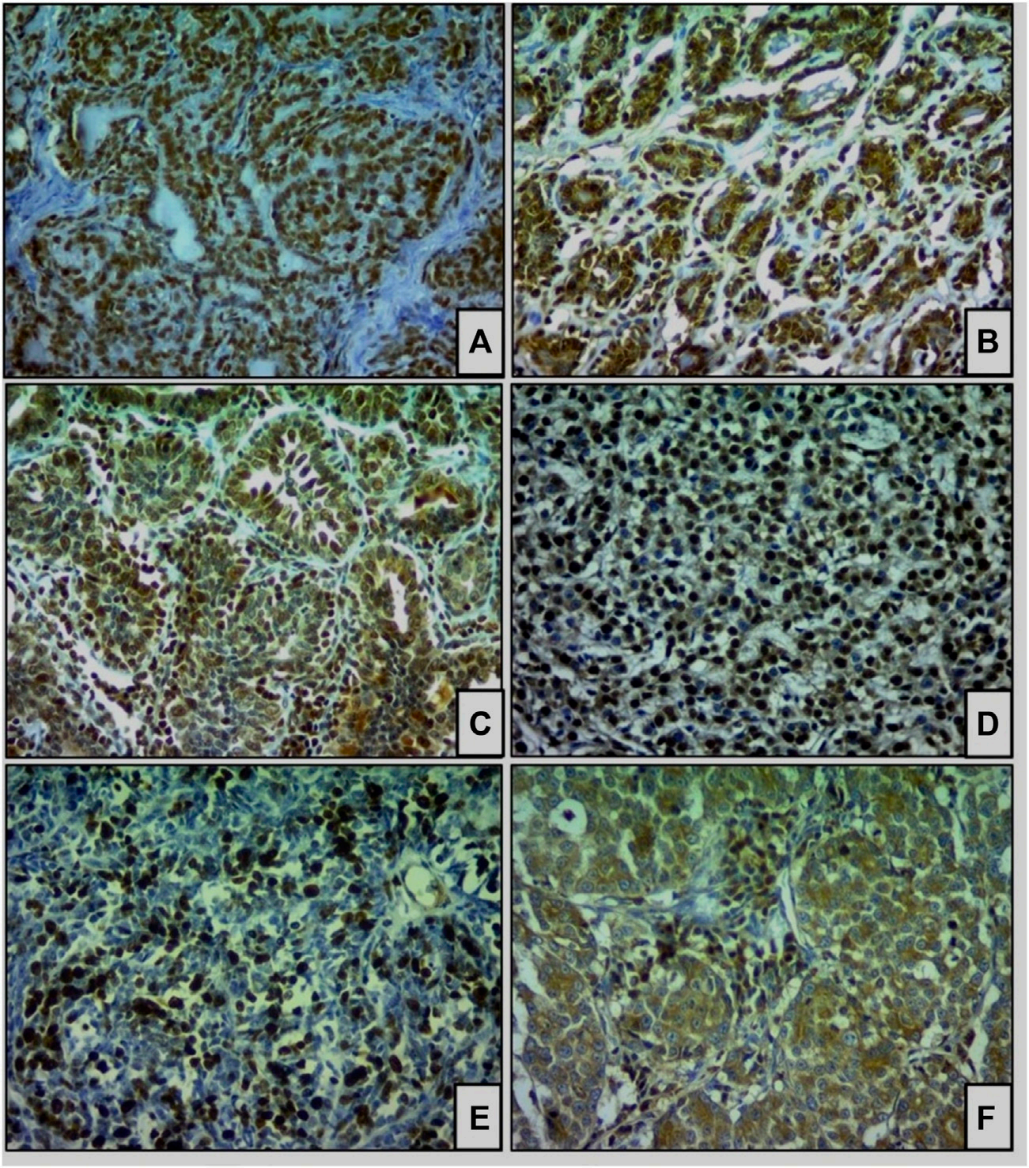
Immunohistochemical staining of acetylated histones and deacetylase enzymes (HDACs) in simple carcinomas (×400). **(A)** H3K9Ac, **(B)** H4K12Ac, **(C)** HDAC1, **(D)** HDAC2, and **(E)** Ki67 are expressed in the nuclei of tumor cells. Staining for **(F)** HDAC6 was observed predominantly in the cytoplasm.

Antibody marking occurred in all groups analyzed. The staining patterns compared with the positive control showed similarities, considering that H3K9Ac, H4K12Ac, HDAC1 and HDAC2 had predominantly nuclear staining, except for HDAC6, which was predominantly cytoplasmic. For Ki67, staining was also observed inside the nucleus (Figure 2), as expected.

All samples of simple breast carcinoma expressed the proteins H3K9Ac, H4K9Ac, HDAC1, and HDAC2 with mean scores of 4.96, 9.86, 5.98, 7.67, respectively, in the immunohistochemical reactions. The median value of the area positive for HDAC6 was 18.7%, adopted as the cut-off value for classifying the samples into low and high expression. The proteins H4K12Ac (54/88.5%), HDAC2 (40/65.6%) and HDAC6 (34/55.7%) exhibited the highest frequency of high expression scores in the analyzed samples. On the other hand, the proteins H3K9Ac (45/73.8%) and HDAC1 (33/54.1%) had the highest frequency of low expression, and these results are described in Table 3.

**TABLE 3:** Immunohistochemical expression of H3K9Ac, H4K12Ac, HDAC1, HDAC2 and HDAC6 in 61 samples of simple mammary carcinoma in female dogs.

Protein	Low expression N° (%)	High expression N° (%)
H3K9Ac	45 (73.8)	16 (26.2)
H4K12Ac	7 (11.5)	54 (88.5)
HDAC1	33 (54.1)	28 (45.9)
HDAC2	21 (34.4)	40 (65.6)
HDAC6	27 (44.3)	34 (55.7)

Pearson chi-square test indicated a low expression of HDAC1 in carcinoma samples of bitches with lymph node metastasis (*p* = 0.035). The other clinicopathological variables were not significantly different concerning the expression of acetylated histones and deacetylase enzymes, as shown in Table 4.

**TABLE 4:** Correlation between the scores obtained for acetylated histones and deacetylase enzymes and the clinicopathological variables of the samples of simple mammary carcinomas in bitches.

Parameters	Total	H3K9Ac			H4K12Ac			HDAC1			HDAC2			HDAC6		
	N°	High	Low	p	High	Low	p	High	Low	p	High	Low	p	High	Low	p
Histological grade		N (%)	N (%)	0.640	N (%)	N (%)	0.862	N (%)	N (%)	0.121	N (%)	N (%)	0.734	N (%)	N (%)	0.943
I	28	6 (21.4)	22 (78.6)		25 (89.3)	3 (10.7)		14 (50.0)	14 (50.0)		18 (64.3)	10 (35.7)		16 (57.1)	12 (42.9)	
II	21	7 (33.3)	14 (66.7)		18 (85.7)	3 (14.3)		10 (47.6)	11 (52.4)		15 (71.4)	6 (28.6)		13 (72.2)	8 (27.8)	
III	12	3 (25.0)	9 (75.0)		11 (91.7)	1 (8.3)		4 (33.3)	8 (66.7)		7 (58.3)	5 (41.7)		7 (58.3)	5 (41.7)	
Histological type				0.511			0.621			0.121			0.08			0.773
Papillary	27	6 (22.2)	21 (77.8)		24 (88.9)	3 (11.1)		12 (44.4)	15 (55.6)		17 (63.0)	10 (37.0)		17 (63.0)	10 (37.0)	
Tubular	24	8 (33.3)	16 (66.7)		22 (91.7)	2 (8.3)		14 (58.3)	10 (41.7)		19 (79.2)	5 (20.8)		14 (58.3)	10 (41.7)	
Solid	10	2 (20.0)	8 (80.0)		8 (80.0)	2 (20.0)		2 (20.0)	8 (80.0)		4 (40.0)	6 (60.0)		5 (50.0)	5 (50.0)	
Size				0.363			0.707			0.284			0.786			0.424
<3 cm	42	9 (21.4)	33 (78.6)		36 (85.7)	6 (14.3)		21 (50.0)	21 (50.0)		26 (61.9)	16 (38.1)		23 (54.8)	19 (45.2)	
≥3 cm	19	7 (33.3)	14 (66.7)		19 (90.5)	2 (9.5)		7 (33.3)	14 (66.7)		14 (66.7)	7 (33.3)		14 (66.7)	7 (33.3)	
mitotic index				0.768			0.404			0.426			0.778			1
0–9/10 HPF	40	10 (25.0)	30 (75.0)		34 (85.0)	6 (15.0)		20 (50.0)	20 (50.0)		27 (67.5)	13 (32.5)		24 (60.0)	16 (40.0)	
>9/10 HPF	21	6 (28.6)	15 (71.4)		20 (95.2)	1 (4.8)		8 (38.1)	13 (61.9)		13 (61.5)	8 (38.1)		12 (57.1)	9 (42.9)	
%Ki67				0.56			0.428			1			0.602			0.435
<18%	32	7 (21.9)	25 (78.1)		27 (84.4)	5 (15.6)		15 (46.8)	17 (53.1)		22 (68.8)	10 (31.2)		17 (53.1)	15 (46.9)	
≥18%	29	9 (31.0)	20 (69.0)		27 (93.1)	2 (6.9)		13 (44.8)	16 (55.2)		18 (62.1)	11 (37.9)		19 (65.5)	10 (34.5)	
Metastasis				0.312			0.670			0.035*			0.074			1
Absent	46	14 (30.4)	32 (69.6)		40 (87.0)	6 (13.0)		25 (54.4)	21 (45.6)		31 (67.4)	15 (32.6)		27 (58.7)	19 (41.3)	
Present	15	2 (13.3)	13 (86.7)		14 (93.3)	1 (6.7)		3 (20.0)	12 (80.0)		6 (40.0)	9 (60.0)		9 (60.0)	6 (40.0)	

*p > 0.05.

**FIGURE 3:**
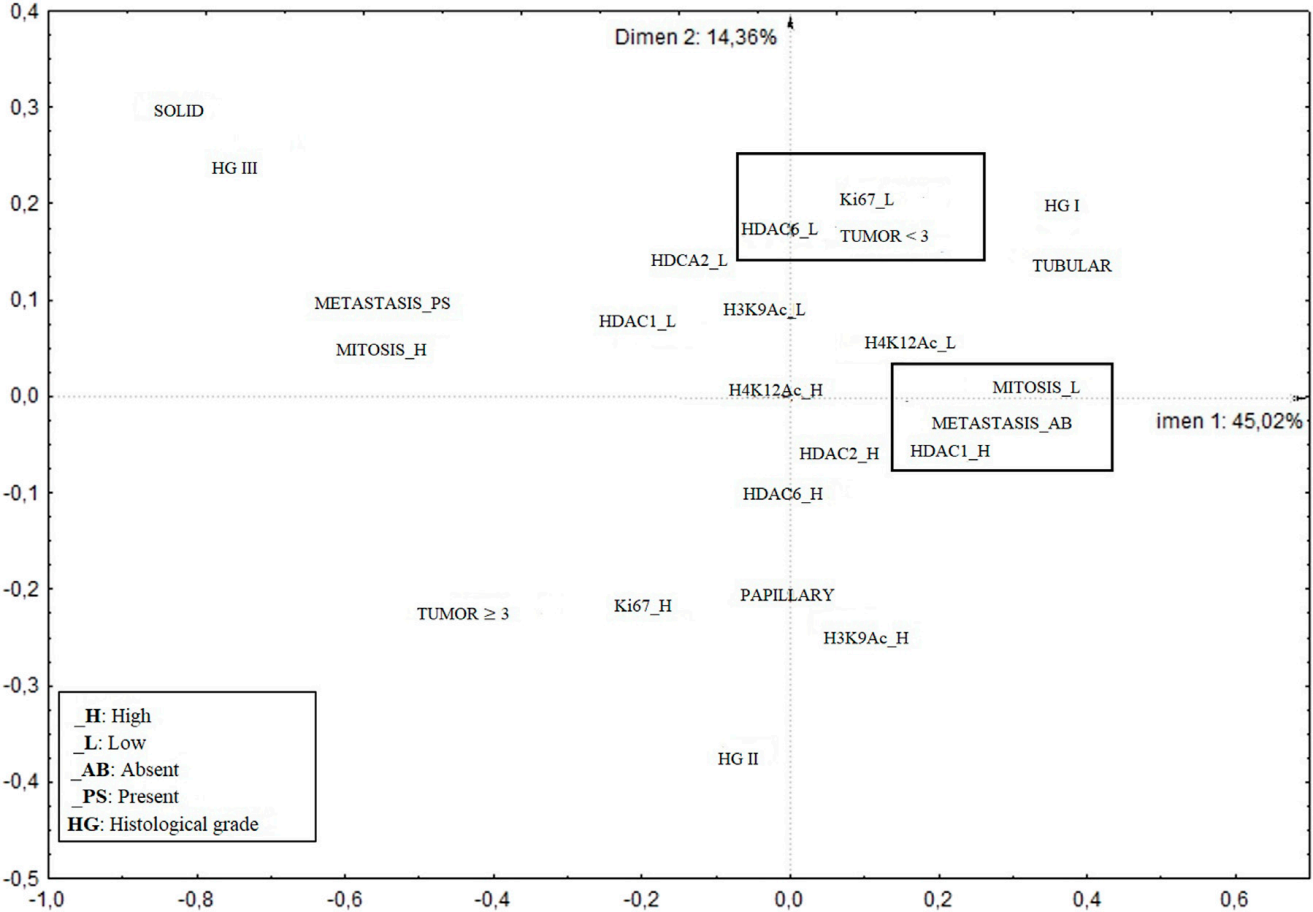
Multivariate correspondence analysis of the associations between the expression of acetylated histones (H3K9Ac and H4K12Ac) and deacetylase enzymes (HDAC 1, 2 and 6) and the clinicopathological variables in canine simple mammary carcinomas.

The evaluation by multivariate correspondence analysis of the clinicopathological variables and the expression of acetylated histones and deacetylase enzymes shows an association between high expression of HDAC1, absence of metastasis, and low mitotic index. There was also an association between low HDAC6 expression and tumors with low Ki67 expression and smaller than 3.0 cm (Figure 3).

### Correlation between acetylated histones and HDACs with survival time

**FIGURE 4:**
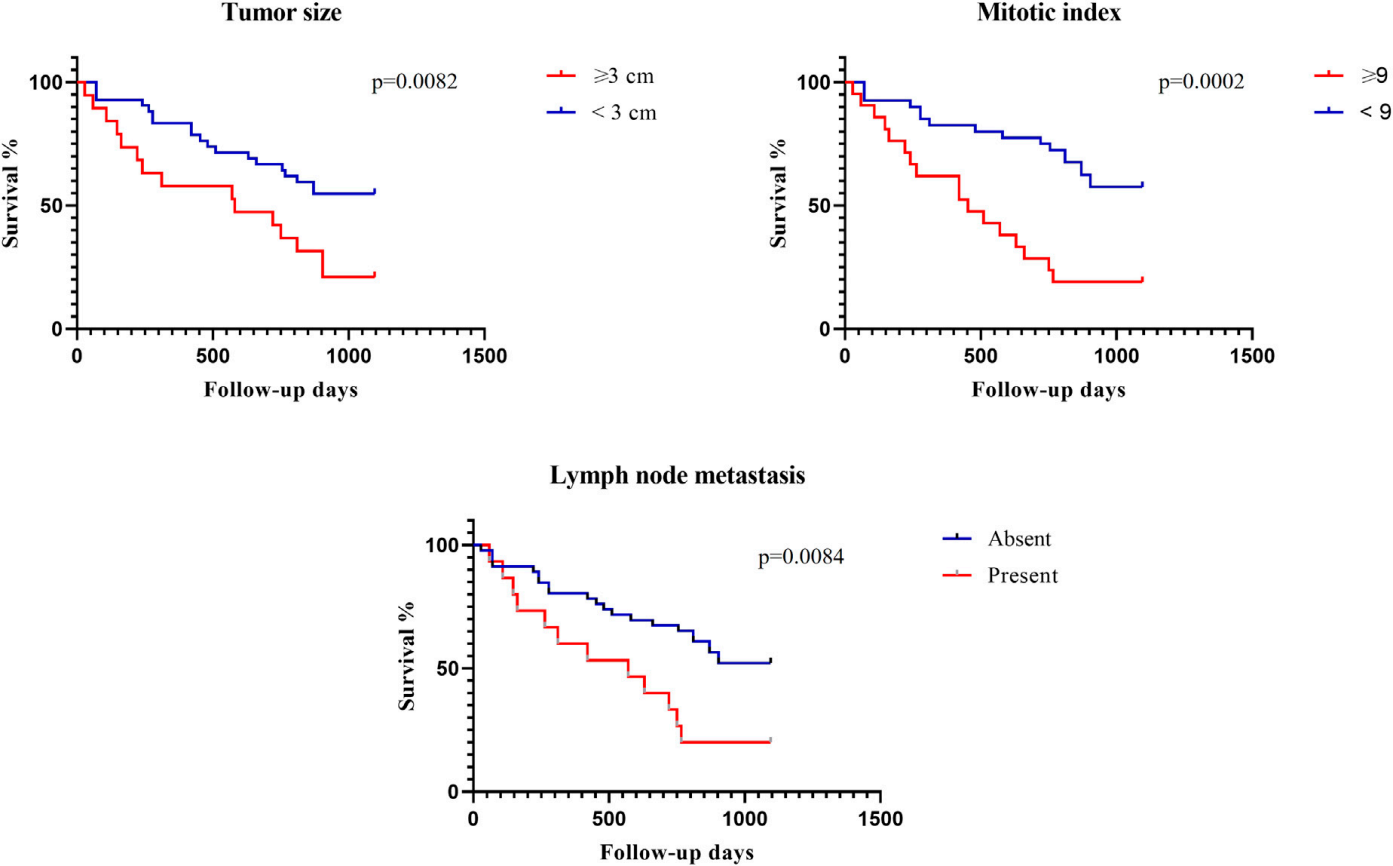
Clinicopathological variables and overall survival time of bitches with simple mammary carcinomas by the Kaplan-Meier method.

Among the clinical-pathological parameters evaluated, traditional prognostic factors such as tumor size (*p* = 0.008), mitotic index (*p* = 0.0002), and lymph node metastasis (*p* = 0.0084) were significantly correlated with the overall survival time (Figure 4). There was no difference for the variables histological grade (*p* = 0.282), histological type (*p* = 0.917) and Ki67 expression (*p* = 0.096).

**FIGURE 5:**
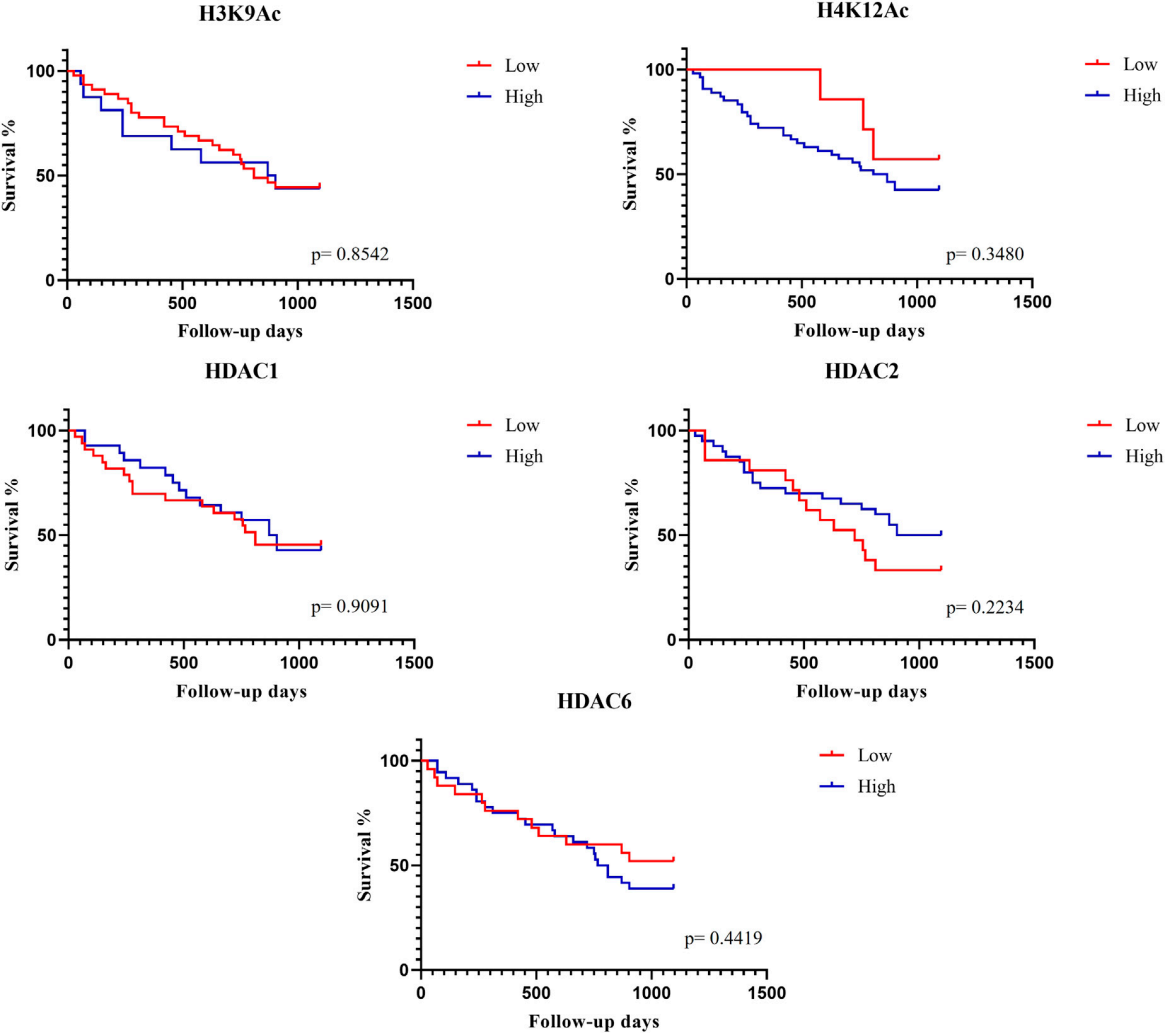
Expression of acetylated histones and deacetylase enzymes concerning overall survival time by the Kaplan-Meier method.

Regarding the expression of acetylated histones and deacetylase enzymes, there was no difference in the overall survival time for H3K9Ac (*p* = 0.8542), H4K12Ac (*p* = 0.3480), HDAC1 (*p* = 0.9091), HDAC2 (*p* = 0.2234), and HDAC6 (*p* = 0.4419) (Figure 5).

## Discussion

Several authors have investigated the epigenetic alterations caused by the expression of acetylated histones and deacetylase enzymes in breast tumors of women and reported that the results were correlated with prognosis (Zhang et al., 2004; Elsheikh et al., 2009; Derr et al.,2014; Zhao et al., 2016). However, this is the first study to investigate the little-known behavior of acetylated histones and deacetylase enzymes in mammary tumors of female dogs, especially in simple mammary carcinomas, and their relationship with the prognosis.

Our research group is also evaluating the expression of acetylated histones and deacetylase enzymes in simple mammary carcinomas and in non-neoplastic mammary tissues of female dogs (data not yet published). Our data suggest aberrant expressions of some of them in neoplastic tissues compared to non-neoplastic mammary tissues without histopathological changes. Like humans, these results show that epigenetic alterations are also identified in mammary tumors in dogs. Our study reveals a high expression of H4K12Ac in 88.5% of the samples analyzed compared to a previous study, in which a high expression of H4K12Ac was observed in 45.1% of the 880 samples of woman breast carcinomas analyzed (Elsheikh et al., 2009). Other researchers have also observed H4K12Ac hypoacetylation in invasive ductal carcinomas (Suzuki et al., 2009). The fact that we did not find H4K12Ac hypoacetylation opens the door for future studies to evaluate other indications of histone acetylation that may be involved in gene transcription, such as H4K16Ac, H4K8Ac, and H4K5Ac (Bernstein, et al., 2005; Fraga et al., 2005; Fraga et al. al., 2005; Pokholok et al., 2005). And, likewise, to compare with healthy tissues to better understand the importance of this finding.

Also, we observed no significant associations between H4K12Ac expression and the clinicopathological variables typically regarded as prognostic factors, nor did we find any correlation with overall patient survival. Conversely, in the case of women with ductal carcinomas, elevated H4K12Ac expression is linked to more favorable histological types, while low expression is associated with high-grade tumors, illustrating a clear connection between acetylation and prognostic indicators (Elsheikh et al., 2009).

Furthermore, we observed a higher percentage of samples with high expression of deacetylase enzymes (HDAC2 and HDAC6) by immunohistochemical analysis. Seo et al. (2014) reported similar observations in 300 samples of breast carcinomas from women. Likewise, moderate and high expressions of HDAC2 were also more frequently observed in breast carcinomas in women (Muller et al., 2013), as well as HDAC6 in human prostatic carcinomas (Hou et al., 2015). These results point to a similarity between the expression of these enzymes in simple mammary carcinomas of dogs and human carcinomas while this information can be useful for future research with therapeutic targets using iHDACs.

By multivariate correspondence analysis, we found an association between low HDAC6 expression and prognostic factors such as low Ki67 expression and tumors smaller than 3.0 cm. The association between low Ki67 expression and smaller tumors represents better prognostic factors (Gizinski et al., 2003; Santos et al., 2013; Nowak et al., 2015), with tumor size as an independent prognostic factor (Sorenmo, 2003; Cavalcanti et al., 2006). The association of HDAC6 expression with the mentioned factors suggests that its expression is related to prognosis in bitches with simple mammary carcinomas and may be a useful antibody in the prognosis.

The HDAC2 and HDAC6 expressions were not correlated with prognostic factors by univariate analysis while the expression of these enzymes had no impact on overall survival as well. Likewise, several studies have evaluated the correlation between overall survival time and HDAC2 expression in breast carcinomas of women and reported no significant difference in the survival time of patients (Muller et al., 2013; Derr et al., 2014). However, other studies in humans associated the expression of HDAC2 with some prognostic factors such as histological grade, presence of metastasis in lymph nodes, and clinical stage of the tumor (Muller et al., 2013; Zhao et al., 2016). In general, high HDAC2 expression was associated with negative prognostic factors and had an impact on overall survival (Zhao et al., 2016).

The H3K9Ac and HDAC1 were among the proteins that had lower expressions in neoplastic tissues in more than half of the samples. These results for H3K9Ac are like those observed in humans, given that studies have shown hypoacetylation in neoplastic tissues (Elsheikh et al., 2009; Webber et al., 2017). Reaffirming these findings, studies in dogs have also demonstrated the same results in urothelial carcinomas (Eto et al., 2019) and more recently in canine cutaneous lymphomas (Matiz, 2019).

Researchers have observed that the hypoacetylation found in many malignant neoplasms is closely linked to the silencing of tumor suppressor genes since hyperacetylation positively influences gene transcription (Taunton, et al., 1996; Kouzarides, 2007; Jones et al., 2016), and possibly the same may be occurring in the canine neoplasms studied.

The expression of HDAC1 in this study was low in most carcinomas. Additionally, the low expression was associated with the presence of metastasis in lymph nodes by univariate analysis. However, also by multivariate analysis, when high expression was associated with favorable prognostic factors (Sorenmo, 2003; Cassali et al., 2014; Soremno et al., 2020), such as absence of metastasis and low mitotic index. These results become even more important considering that the increasingly accurate prognosis in bitches with breast tumors is aligned with the results reported for women with breast cancer. A meta-analysis evaluating seven papers reporting on the HDAC1 expression in breast tumors in women found that the higher expression of this enzyme is associated with favorable prognostic factors and better overall survival times (Qiao et al., 2018).

In our study, we were able to reaffirm the role of some clinicopathological variables in the prognosis of patients. Variables such as tumor size, presence of metastasis, and mitotic index significantly influenced the overall survival of bitches. These results are in agreement with those observed in other studies that evaluated prognostic factors in mammary neoplasms in bitches (Misdorp, 1999; Ferreira et al., 2009; Santos et al., 2013; Rasotto et al., 2017), and reaffirm the importance of these criteria in the decision-making process and clinical approach to treating patients.

In general, we were not able to determine the impact of the expression of acetylated histones and deacetylase enzymes on the overall survival time of bitches, and we also did not observe an association with some of the clinicopathological variables. These results may be related to the number of samples analyzed compared to human studies. Thus, we believe that further studies investigating a considerably higher number of samples may clarify further the relationship between prognosis and epigenetic changes in mammary tumors in bitches.

Furthermore, understanding the epigenetic changes involved in mammary neoplasms in bitches is of considerable importance, because in addition to the prognostic factors that can be incorporated into the assessment of simple mammary carcinomas, the high expressions of HDAC2 and HDAC6 enzymes may, in the future, become therapeutic targets in this neoplasm.

The HDAC inhibitors are already being incorporated into cancer treatment in humans (Moufarrij et al., 2020) and several studies show that the modulation of the expression of these enzymes favors the immune system and the transcription of tumor suppressor genes (Lansigan and Foss, 2010; Fang et al., 2015; Lu et al., 2020). Thus, our results support a possible use in dogs as well. In addition, phase I clinical studies testing the toxicity of iHDACs have already been performed and have shown good results (Zhang et al., 2016).

Our results are pioneering concerning the evaluation of acetylated histones and deacetylase enzymes in bitches with simple mammary carcinomas and point to a relationship between high expression of HDAC1 and low HDAC6 with favorable prognostic factors. Moreover, the low expression of H3K9Ac and the high expressions of HDAC2 and HDAC6 support the continuity of research involving these enzymes as therapeutic targets. The expansion of this study with a greater number of samples may confirm the results described here.

## Data Availability

The original contributions presented in the study are included in the article/Supplementary Material further inquiries can be directed to the corresponding author.
